# Icariin Inhibits AGE-Induced Injury in PC12 Cells by Directly Targeting Apoptosis Regulator Bax

**DOI:** 10.1155/2019/7940808

**Published:** 2019-04-22

**Authors:** Shao-Yang Zhao, Li-Xi Liao, Peng-Fei Tu, Wei-Wei Li, Ke-Wu Zeng

**Affiliations:** ^1^Research Studio of Integration of Traditional and Western Medicine, First Hospital, Peking University, Beijing 100034, China; ^2^State Key Laboratory of Natural and Biomimetic Drugs, School of Pharmaceutical Sciences, Peking University, Beijing 100191, China

## Abstract

Diabetic encephalopathy (DE) is a serious complication caused by long-term cognitive impairment in diabetic patients. At present, there is no effective treatment for DE. Icariin (ICA) is a bioactive ingredient isolated from *Epimedium*. Previous research indicated that ICA was neuroprotective against A*β*-induced PC12 cell insult; however, the effect of ICA on an advanced glycosylation end product- (AGE-) induced neural injury model has not been studied. In this study, we investigated the neuroprotective effects of ICA on AGE-induced injury in PC12 cells. Our findings revealed that ICA could effectively protect PC12 cells from AGE-induced cell apoptosis by suppressing oxidative stress. Moreover, we observed that ICA could significantly protect against mitochondrial depolarization following AGE stimulation and inactivate the mitochondria-dependent caspase-9/3 apoptosis pathway. Most notably, we identified the direct target protein of ICA as apoptosis regulator Bax by a pulldown assay. We found that ICA could specifically target Bax protein and inhibit Bax dimer formation and migration to mitochondria. Furthermore, a siRNA knockdown experiment revealed that ICA could inhibit PC12 cell apoptosis and oxidative stress through targeting Bax. Taken together, our findings demonstrated that ICA could attenuate AGE-induced oxidative stress and mitochondrial apoptosis by specifically targeting Bax and further regulating the biological function of Bax on mitochondria.

## 1. Introduction

Diabetes mellitus (DM) is a common metabolic disease. In the development of DM, a variety of complications can be formed, of which diabetic encephalopathy (DE) is a major central nervous system complication. The main features of DE include cognitive dysfunction and neurodegeneration [1–3]. Although the pathogenesis of DE is still not clear, recent studies have found that overproduction of advanced glycosylation end products (AGEs) is closely related to the occurrence of DE [4, 5]. AGEs are toxic substances produced by nonenzymatic glycosylation of proteins and reducing sugars under long-term hyperglycemia. Therefore, it is particularly important to develop drugs that inhibit oxidative stress induced by AGEs in neurons. The accumulation of AGEs in the brain can mediate progressive neurostructural changes and functional deficits through different pathways [6–9]. AGEs can induce oxidative stress and reactive oxygen species (ROS) formation, leading to cell damage and even apoptosis [10–12].

A growing number of studies have shown that oxidative stress can occur in the course of a variety of neurodegenerative diseases, such as Alzheimer's disease, Parkinson's disease, and stroke [13–15]. Under normal conditions, the generation and elimination of ROS in vivo are in a dynamic equilibrium state. When the body is stimulated by certain factors including AGEs, oxygen-glucose deprivation/reperfusion (OGD/R), radiation, tumor, smoking, and alcohol, it will cause the body to produce excessive ROS. Excessive ROS can activate a series of cascade reactions, such as caspase signaling pathway, and ultimately lead to neuronal apoptosis. Meanwhile, an excess amount of ROS can cause a decrease in the mitochondrial membrane potential and the opening of the mitochondrial permeability transition pore (mtPTP), allowing cytochrome C to be released from the mitochondria. Then, cytochrome C can bind to apoptosis activating factor-1 (Apaf-1), activate the downstream caspase signaling pathway, and initiate apoptosis [16]. In addition, studies have shown that proapoptotic Bcl-2 family proteins, such as Bax, can promote the opening of mtPTP and activate the caspase cascade [17].

Icariin (ICA) is an active ingredient extracted from the traditional Chinese medicine *Epimedium*. A large number of studies have confirmed that ICA can significantly inhibit neuronal apoptosis, increase neuron viability, delay neuropathological process, and improve neuronal function [18–20]. Moreover, ICA has a broad application in the development of neuroprotective drugs. However, the potential pharmacological mechanism of ICA is still unknown. Therefore, in this study, we tried to explore the neuroprotective effects of ICA on AGE-induced PC12 cells as well as the potential pharmacological mechanism.

## 2. Materials and Methods

### 2.1. Materials

**Figure 1:**
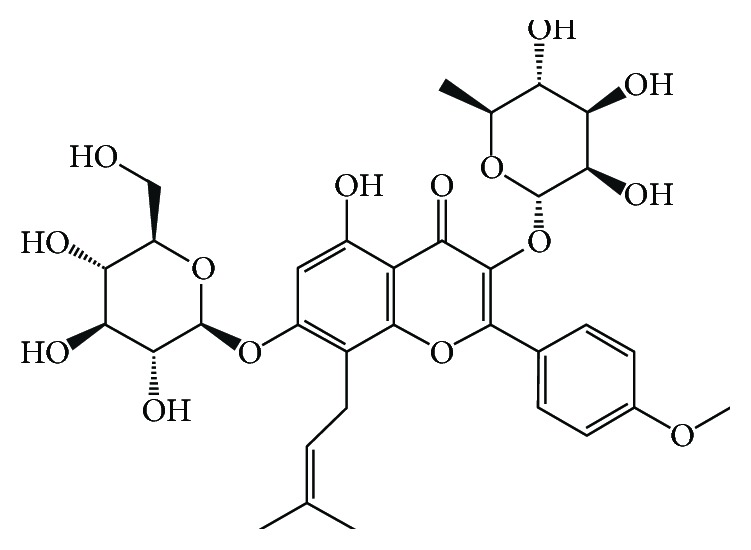
The chemical structure of Icariin (ICA).

Icariin (ICA, C_33_H_40_O_15_) was obtained from Chengdu Push Bio-Technology Co. Ltd. (Chengdu, China) with a purity greater than 98% by HPLC. The structure of ICA is shown in Figure 1. Advanced glycosylation end products (AGEs) were purchased from Beijing Biosynthesis Biotechnology Co. Ltd. (Beijing, China) with a purity greater than 95% by HPLC. Dulbecco's Modified Eagle's medium (DMEM) and fetal bovine serum (FBS) were purchased from PAN-Biotech GmbH (Aidenbach, Germany). 3-(4,5-Dimethylthiazol-2-yl)2,5-diphenyltetrazolium bromide (MTT) was from Sigma-Aldrich Chemical Co. (Saint Louis, MO, USA). A lactate dehydrogenase (LDH) assay kit, superoxide dismutase (SOD) kit, and malondialdehyde (MDA) kit were purchased from Nanjing Jiancheng Bioengineering Institute (Nanjing, China). Hoechst 33258 and an acridine orange (AO)/ethidium bromide (EB) double-staining kit were obtained from Beijing Solarbio Science & Technology Co. Ltd. (Beijing, China). A mitochondrial membrane potential assay kit with JC-1 and 2,7-dichlorofluorescein diacetate (DCFH-DA) were from Beyotime Institute of Biotechnology (Nanjing, China). The MitoSOX Red mitochondrial superoxide indicator and Lipofectamine RNAiMAX were purchased from Invitrogen® (Thermo Fisher Scientific, Waltham, MA, USA). The Opti-MEM reduced serum medium was from Gibco® (Thermo Fisher Scientific, Waltham, MA, USA). Recombinant human Bax, FAM3C, and plasminogen receptor proteins were purchased from Abcam (Massachusetts, US). Recombinant human prohibitin and prohibitin-2 were purchased from ProSpec-Tany TechnoGene Ltd. (Ness Ziona, Israel). Enhanced BCA protein assay kit (TransGen Biotech Co. Ltd., Beijing, China). Primary antibodies for caspase-3, cleaved caspase-3, PARP, cleaved PARP, Bax, *α*-tubulin, COX-IV, and rabbit IgG were obtained from Cell Signaling Technology (Boston, MA, USA).

### 2.2. Cell Culture

Rat PC12 pheochromocytoma cells were purchased from the Cell Center of the Chinese Academy of Medical Sciences (Beijing, China). The PC12 cells were cultured in DMEM containing 10% (*v*/*v*) heat-inactivated FBS, 100 U/mL penicillin, and 100 *μ*g/mL streptomycin at 37°C in a 5% CO_2_ humidified incubator.

### 2.3. Sample Treatment

The cells were divided into the control group (only DMEM), the model group (300 *μ*g/mL AGEs), the low-concentration ICA treatment group (300 *μ*g/mL AGEs with 5 *μ*M ICA), the medium-concentration ICA treatment group (300 *μ*g/mL AGEs with 10 *μ*M ICA), and the high-concentration ICA treatment group (300 *μ*g/mL AGEs with 20 *μ*M ICA). Firstly, all groups were serum starved for 24 h and then replaced with normal cell culture medium. Then, the model group was stimulated with AGEs (300 *μ*g/mL) and the ICA treatment groups were cotreated with ICA (5, 10, and 20 *μ*M) and AGEs (300 *μ*g/mL) for 48 h.

### 2.4. Cell Viability Assay

PC12 cells were seeded into a 96-well plate at a density of 3500/well for 24 h. Then, the cells were grouped and treated with AGEs (300 *μ*g/mL) and ICA (5, 10, and 20 *μ*M) as mentioned previously. The cell viability was assayed by MTT. In brief, the original medium was removed and the MTT solution was added (0.5 mg/mL) for 4 h at 37°C. Then, cells were dissolved in 200 *μ*L DMSO and the formazen absorbance was measured at 570 nm with a microplate reader (Bio-Rad Laboratories Inc., Hercules, CA, USA). The relative cell viability was expressed as the percentage of control.

### 2.5. Lactate Dehydrogenase (LDH) Assay

For the LDH assay, PC12 cells were stimulated with AGEs (300 *μ*g/mL) and ICA treatment (5, 10, and 20 *μ*M) as described previously. Then, the culture supernatant was collected and analyzed using a commercial LDH kit according to manufacturer's instructions. The absorbance was detected at 450 nm.

### 2.6. Hoechst 33258 Staining and AO/EB Staining

The cells were seeded into a 24-well plate at a density of 20000/well for 24 h and subjected to AGE (300 *μ*g/mL) insult and ICA treatment (5, 10, and 20 *μ*M) as described previously. The medium was removed and 4% paraformaldehyde was added for 20 min. Then, the cells were stained with Hoechst 33258 solution (1 *μ*g/mL) in the dark for 30 min at room temperature. Images were captured using a fluorescence microscope (IX73, Olympus, Japan) under an excitation wavelength of 352 nm and an emission wavelength of 461 nm [21, 22].

For AO/EB staining, PC12 cells were seeded into a 48-well plate at a density of 8000/well for 24 h and stimulated with AGEs (300 *μ*g/mL) and ICA treatment (5, 10, and 20 *μ*M) as described previously. After removing the culture medium, PC12 cells were added into the AO/EB mixture (100 *μ*g/mL AO and 100 *μ*g/mL EB mixed) for 8 min in the dark at room temperature. Then, the cells were washed three times with PBS and observed using a fluorescence microscope under an excitation wavelength of 490 nm and an emission wavelength of 530 nm for AO staining and an excitation wavelength of 520 nm and an emission wavelength of 590 nm for EB staining [22].

### 2.7. Detection of Superoxide Dismutase (SOD) and Malondialdehyde (MDA)

The SOD and MDA productions were measured by commercial kits. Briefly, PC12 cells were treated with AGEs (300 *μ*g/mL) and ICA (5, 10, and 20 *μ*M) for 48 h as described before. Then, PC12 cells were lysed and the supernatants were collected. The levels of SOD and MDA were determined according to the manufacturer's instructions.

### 2.8. Detection of Reactive Oxygen Species (ROS)

For intracellular ROS detection, PC12 cells were seeded into 6-well plates at a density of 5 × 10^5^ and treated with AGEs (300 *μ*g/mL) and ICA (5, 10, and 20 *μ*M) for 24 h. PC12 cells were washed with serum-free DMEM for 3 times and stained with 10 *μ*Μ DCFH-DA, a specific fluorescent probe at 37°C for 30 min in the dark. For MitoSOX Red staining, PC12 cells were seeded into a 48-well plate at a density of 8000/well for 24 h and treated as described previously. The cells were washed with PBS for three times and incubated with 5 *μ*M MitoSOX working solution for 10 min at 37°C in the dark. Then, ROS staining was observed under a fluorescence microscope.

### 2.9. Mitochondrial Membrane Potential Assay

Mitochondrial depolarization was assessed using a commercial JC-1 assay kit. After having been treated with AGEs (300 *μ*g/mL) and ICA (5, 10, and 20 *μ*M), the cells were incubated with the JC-1 working solution (10 *μ*g/mL) at 37°C for 15 min in the dark. After washing with the JC-1 buffer solution, the images were captured using a fluorescence microscope under an excitation wavelength of 460 nm and an emission wavelength of 530 nm for the JC-1 monomer and an excitation wavelength of 520 nm and an emission wavelength of 590 nm for the JC-1 polymer.

### 2.10. Western Blot Analysis

PC12 cells were collected after being treated with AGEs (300 *μ*g/mL) and ICA (5, 10, and 20 *μ*M) for 48 h. The cells were lysed in cold RIPA with a cocktail protease inhibitor (1x) for 30 min. The supernatant was collected, and protein concentrations were determined by an enhanced BCA protein assay kit. Cytosol- and mitochondria-enriched fractions were isolated with the Mitochondria Isolation Kit (Pierce, Rockford, USA) for Bax analysis. For other proteins, total cell lysates were separated by SDS-PAGE (10%-15%) and transferred to polyvinylidene fluoride (PVDF) membranes. The PVDF membranes were then blocked with 5% nonfat milk and incubated overnight with the indicated primary antibodies (1 : 1000) at 4°C. The membranes were washed with PBST (phosphate-buffered saline, 0.1% Tween 20) for three times and incubated with secondary antibody at room temperature for 2 h. Then, the membranes were washed with PBST for another three times and visualized using an enhanced chemiluminescent substrate and scanned with the Tanon 5200 Imaging Analysis System (Tanon Science & Technology Co. Ltd., Shanghai, China). Relative protein expressions were shown by densitometry analysis using Gel-Pro analyzer 4 software.

### 2.11. Pulldown of Direct Target Proteins of ICA

The ICA target proteins were identified using the pulldown method according to a previous report with a slight modification [23]. Briefly, ICA was coupled to the Epoxy-Activated Sepharose 6B beads (GE Healthcare Life Sciences, Chicago, IL, USA). The vehicle beads and ICA-coupled beads were subsequently mixed with cell lysates and incubated overnight at 4°C. The bead-captured proteins were obtained and separated to 10%-SDS-PAGE, visualized by silver staining, and identified by LC-MS/MS using a nano-HPLC-tandem LTQ-Orbitrap Velos Pro mass spectrometer (LC-MS/MS) as previously reported [24].

### 2.12. Surface Plasmon Resonance (SPR) Assay

Interaction between ICA and potential target proteins (Bax, FAM3C, Prohibitin, Prohibitin-2, and Plasminogen receptor) were, respectively, analyzed using the Biacore T200 system (GE Healthcare Life Sciences). The recombinant human proteins were immobilized on a carboxymethylated 5-sensor chip using a standard amine-coupling method. Different concentrations of potential target proteins (in the range of 1.95-50 *μ*M) in the running buffer were injected as analytes at a constant flow rate of 30 *μ*L/min with a contact time of 60 s and a dissociation time of 120 s. The interaction parameters (i.e., association (*k*_*a*_), dissociation (*k*_*d*_), and the equilibrium dissociation constant of the complex (*K*_*D*_)) were analyzed with the Biacore evaluation software (T200 Version 2.0) according to the 1 : 1 Langmuir model.

### 2.13. Drug Affinity Responsive Target Stability (DARTS) Assay

PC12 lysates were added into a TNC buffer with a dilution of 1 : 10 (50 mM Tris·Cl, pH 8.0, 50 mM NaCl, and 50 mM CaCl_2_) and incubated with indicated concentrations of ICA (0, 20, 50, and 100 *μ*M) or DMSO as control. After 4 h of incubation at 4°C, pronase (2 *μ*g/mL) was added into the lysates for a further 15 min at room temperature. Then, SDS-PAGE loading buffer was added into the lysates to halt the reaction at 98°C for 8 min, and the protein bands were detected by immunoblot with a specific anti-Bax antibody [24, 25].

### 2.14. Cellular Thermal Shift Assay (CETSA)

PC12 cells were collected and freeze-thawed five times using liquid nitrogen. The cell lysates were grouped into two aliquots, with one serving as the control and the other being treated with ICA (20 *μ*M) for 4 h at 4°C. Then, the lysates were heated at indicated temperatures (48 and 66°C, respectively) for 5 min followed by cooling at room temperature. The protein bans were detected by immunoblot [24, 26].

### 2.15. Chemical Cross-Linking of Bax

PC12 cells were cultured and stimulated with AGEs (300 *μ*g/mL) and ICA treatment (20 *μ*M) as described previously. The cells were lysed with NP40 buffer and cross-linked with 1 mM disuccinimidyl suberate in buffer (20 mM HEPES, 100 mM KCl, and 1 mM dithiothreitol, pH 8.0) for 1 h at room temperature. The reaction was quenched by adding 50 mM Tris-HCl for 15 min [24]. The samples were analyzed by western blot for Bax. Theoretically, the Bax monomer band is around 21 kDa and the dimer band is around 42 kDa.

### 2.16. Transient Transfection with Bax siRNA

For siRNA knockdown studies, PC12 cells were transfected with Bax or negative siRNA (Suzhou GenePharma Co. Ltd., Suzhou, China). siRNAs were premixed with Lipofectamine RNAiMAX in Opti-MEM reduced serum medium for 48 h. Then, the transfected cells were used for further research.

### 2.17. Statistical Analysis

All values were presented as mean ± S.D. from at least three independent experiments. Statistical analysis was performed by one-way ANOVA followed by Tukey's post hoc test. Differences were considered statistically significant at *P* < 0.05.

## 3. Results

### 3.1. ICA Protected PC12 Cells from AGE-Induced Apoptosis

**Figure 2:**
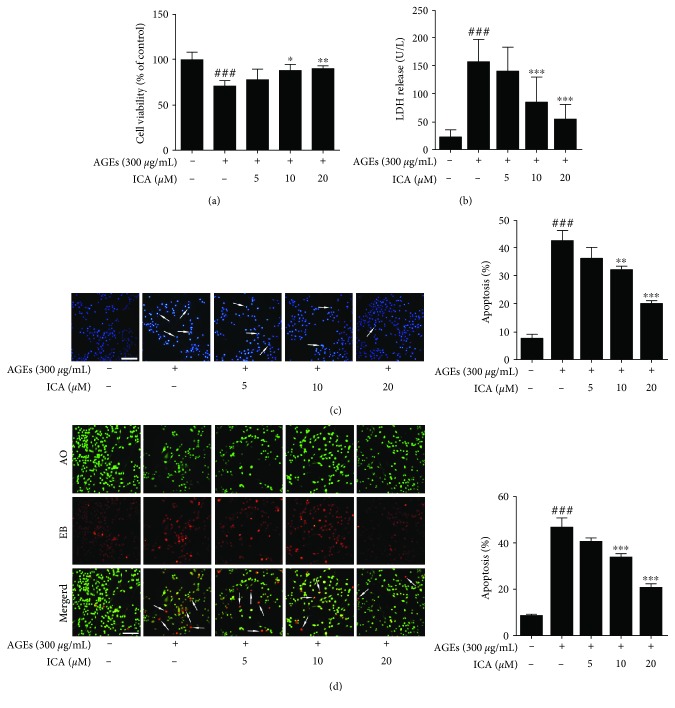
ICA protected PC12 cells against AGE-induced apoptosis. (a) PC12 cells were exposed to AGEs and treated with ICA (5, 10, and 20 *μ*M) for 48 h. Cell viability was assessed by MTT and expressed relative to control. (b) PC12 cells were treated as in (a), and LDH release was detected with a commercial kit. (c) Apoptotic nuclei were identified using Hoechst 33258 staining. Scale bar = 50 *μ*m. (d) Apoptotic cells were identified by double staining with AO and EB. The cells which took up both dyes were classified as apoptotic (indicated by arrows in the merged images). Scale bar = 50 *μ*m. All data were presented as mean ± S.D. from at least three independent experiments. ^###^*P* < 0.001 relative to the control group; ^∗^*P* < 0.05, ^∗∗^*P* < 0.01, and ^∗∗∗^*P* < 0.001 relative to the model group.

In this study, we first investigated whether ICA could protect PC12 cells from AGE insult by using the MTT assay. As shown in Figure 2(a), AGE (300 *μ*g/mL) insult for 48 h caused a significant decrease in the viability compared with the control group (*P* < 0.001), and ICA treatment (5, 10, and 20 *μ*M) could significantly prevent cell death in a concentration-dependent manner (*P* < 0.01 or *P* < 0.001). Moreover, the result was further confirmed by the LDH assay, which could be used as an indicator of cell toxicity. We found that AGE (300 *μ*g/mL) stimulation for 48 h markedly facilitated LDH release from PC12 cells, which was four times as much as the control group (*P* < 0.001). ICA treatment (5, 10, 20 *μ*M) could also significantly inhibit the release of LDH in a concentration-dependent manner (*P* < 0.001) (Figure 2(b)). In addition, Hoechst 33258 staining, a sensitive DNA dye assay for apoptosis, revealed that AGE insult could dramatically induce chromosome condensation (accounting for 44.2% of the total number of cells) (*P* < 0.001), which was a crucial apoptosis phenomenon in cells. ICA effectively attenuated the change (*P* < 0.01 or *P* < 0.001) (Figure 2(c)). To further verify the effect of ICA on cell apoptosis, we used AO/EB double-staining analysis. As depicted in Figure 2(d), the proportion of AO/EB colocalization increased to 54.1% after the induction of AGEs (300 *μ*g/mL) for 48 h (*P* < 0.001), indicating the increased proportion of apoptotic cells. However, the percentage of apoptotic cells significantly decreased upon ICA treatment (5, 10, and 20 *μ*M) in PC12 cells (*P* < 0.001). All of these results suggested that AGEs significantly induced PC12 cell apoptosis, and ICA could protect PC12 cells from AGE insult.

### 3.2. ICA Inhibited Oxidative Stress in PC12 Cells Induced by AGEs

**Figure 3:**
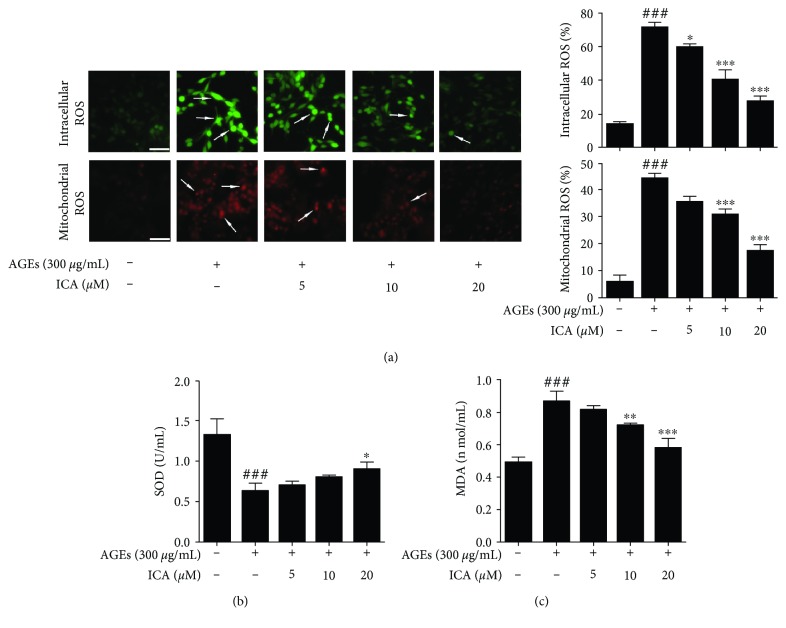
ICA inhibited oxidative stress in AGE-induced PC12 cells. (a) PC12 cells were exposed to AGEs and treated with ICA (5, 10, and 20 *μ*M) for 48 h. Detection of intracellular and mitochondrial ROS using DCFH-DA and MitoSOX. Scale bar = 50 *μ*m. (b and c) PC12 cells were treated as in (a), and the intracellular level of SOD and MDA were detected. Data were presented as mean ± S.D. from independent experiments performed in triplicate. ^###^*P* < 0.001 relative to the control group; ^∗^*P* < 0.05, ^∗∗^*P* < 0.01, and ^∗∗∗^*P* < 0.001 relative to the model group.

It has been reported that AGEs could bind to their cell surface receptor RAGE, leading to the oxidative stress response [27]. Thus, we first detected the production of intracellular and mitochondrial reactive oxygen species (ROS) by DCFH-DA and MitoSox, respectively. The results showed that AGE stimulation significantly increased the intracellular and mitochondrial ROS productions (*P* < 0.001), which were effectively reversed by ICA (*P* < 0.05 or *P* < 0.001) (Figure 3(a)). To further determine the antioxidant activity of ICA in AGE-induced PC12 cells, we next measured the superoxide dismutase (SOD) and malondialdehyde (MDA) productions. The data showed that SOD activity was half of that of the control group (*P* < 0.001). ICA could also increase the activity of SOD in a concentration-dependent manner (*P* < 0.05). Meanwhile, AGE insult increased MDA production in PC12 cells (*P* < 0.001), which was also markedly blocked by ICA treatment (*P* < 0.01 or *P* < 0.001) (Figures 3(b)–3(c)). This result suggested that ICA improved the activity of PC12 cells by scavenging free radicals.

### 3.3. ICA Inhibited PC12 Cell Apoptosis via Mitochondria-Dependent Caspase-9/3 Pathway

**Figure 4:**
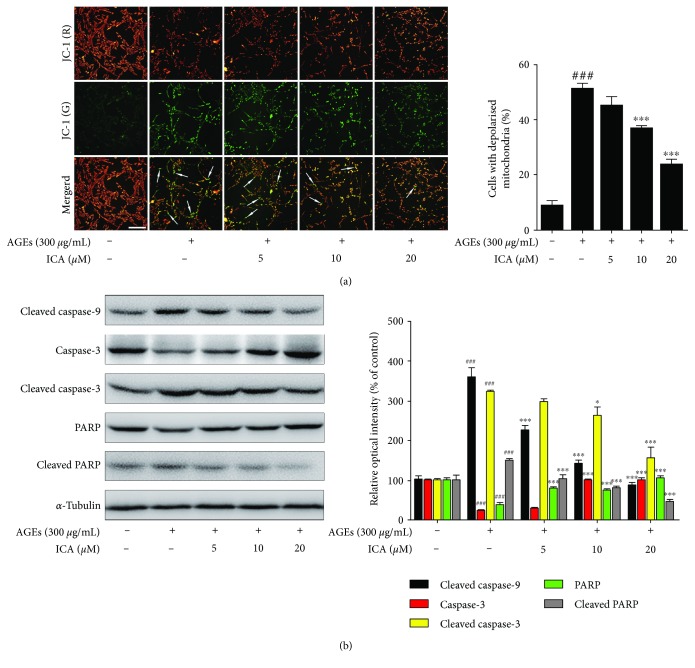
ICA protected PC12 cells against AGE-induced apoptosis via the caspase-9/3 pathway. (a) PC12 cells were subjected to AGEs with or without ICA extract for 48 h. Mitochondrial depolarization was investigated by the JC-1 staining assay. The cells with depolarized mitochondria were identified by green fluorescence. Scale bar = 50 *μ*m. (b) The cells were treated as in (a). The protein levels of caspase-3, PRAR, cleaved caspase-9, cleaved caspase-3, and cleaved PARP were examined by western blot. Data were presented as mean ± S.D. from independent experiments performed in triplicate. ^###^*P* < 0.001 relative to the control group; ^∗^*P* < 0.05 and ^∗∗∗^*P* < 0.001 relative to the model group.

The decline of mitochondrial membrane potential is a landmark event in the early stage of cell apoptosis [28]. Thus, we detected the mitochondrial membrane potential using a potential-sensitive fluorescent probe, JC-1. In normal cells, JC-1 aggregates in the mitochondrial matrix to form a polymer that emits red fluorescence, while in depolarized mitochondria, JC-1 is a monomer that emits green fluorescence [29]. In our study, AGEs promoted a dramatic increase in the number of cells with depolarized mitochondria (green), which accounted for 56.7% of the total number of cells. This increase was also reversed by concentration-dependent ICA treatment (*P* < 0.001) (Figure 4(a)). When mitochondrial dysfunction occurs, the mitochondria-dependent caspase signaling pathways can be involved in cell apoptosis subsequently. Therefore, we determined the protein expressions of the caspase-9/3 signaling pathway. Upon the stimulation of AGEs, the levels of cleaved caspase-9, cleaved caspase-3, and cleaved PARP were significantly increased, whereas the levels of procaspase-3 and PARP were decreased compared with the normal group. These effects were also significantly reversed by different concentrations of ICA (Figure 4(b)).

### 3.4. ICA Directly Targeted Apoptosis Regulator Bax

**Figure 5:**
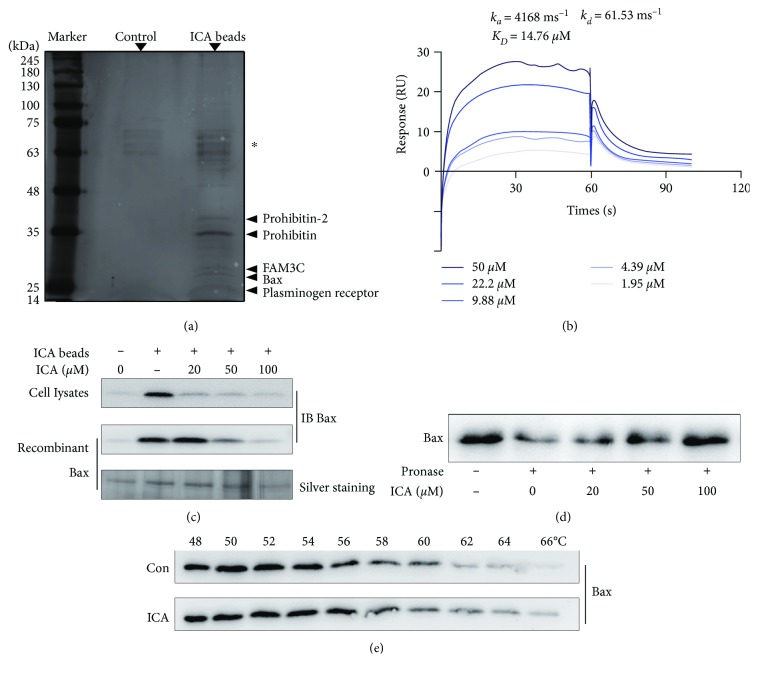
Bax is a direct target protein for ICA. (a) The PC12 lysates were incubated with ICA beads or control beads, and then the potential proteins bound to the beads were resolved by SDS-PAGE, followed by silver staining. The asterisk-marked bands indicated nonspecific bead-bound vimentin and actin. (b) SPR analysis of ICA binding to Bax. The kinetic parameters of *k*_*a*_, *k*_*d*_, and *K*_*D*_ were derived by fitting to a 1 : 1 Langmuir binding model. (c) ICA beads were incubated with PC12 lysates or recombinant Bax protein in the absence or presence of ICA for competitive binding, and the proteins bound to the beads were analyzed by western blot or silver staining. (d) ICA promoted Bax resistant to pronase (DARTS). PC12 lysates were incubated with pronase in the absence or presence of ICA, and Bax was detected by western blot. (e) PC12 lysates were incubated with ICA (20 *μ*M) or vehicle followed by the cellular thermal shift assay (CETSA). Data were presented as mean ± S.D. from independent experiments performed in triplicate.

Drug targets are the major biological basis for treating various diseases. Thus, it is important for deeply interpreting the pharmacological action mechanism of ICA by exploring its potential pharmacological targets. Here, we prepared ICA-conjugated epoxy-activated sepharose beads (ICA beads) as an affinity reagent to capture the target proteins. Thus, we captured the potential target proteins from PC12 lysates by using ICA beads. Then, the proteins bound to beads were separated by SDS-PAGE and stained with silver. Compared with the control group, five obvious protein bands (15-35 kDa) were identified as plasminogen receptor, Bax, FAM3C, prohibitin, and prohibitin-2 by LC-MS/MS analysis (Figure 5(a)).

To analyze the interaction of ICA with these five potential proteins, we used SPR technology for quantitative investigation. As shown in Figure 5(b), ICA specifically bound to Bax with an equilibrium dissociation constant (*K*_*D*_) value of 14.76 *μ*M, indicating that ICA had a strong binding capacity to Bax. However, the binding effects of the other four proteins were not specific based on SPR, which was excluded from the alternative targets of ICA. We next performed a pulldown assay to verify the interaction of ICA with Bax. The recombinant Bax protein or PC12 cell lysates were incubated with ICA beads in the absence or presence of an excess amount of ICA for binding competition. The results of western blot and silver staining showed that Bax could be pulled down by ICA beads, while the binding was concentration-dependently blocked by the excess amount of ICA competition (Figure 5(c)).

Small molecules that specifically bind to target proteins could enhance the proteins' stability via forming a ligand-protein complex [30]. Therefore, we next aimed to investigate whether ICA could increase the stability of Bax by the DARTS and CETSA assay. The DARTS showed that ICA could concentration-dependently inhibit Bax degradation induced by pronase (Figure 5(d)). Meanwhile, CETSA showed that ICA could protect Bax from temperature-dependent degradation (Figure 5(e)). All these results suggested that ICA had a direct interaction with Bax.

### 3.5. ICA Inhibited Bax Dimer Formation and Migration to Mitochondria

**Figure 6:**
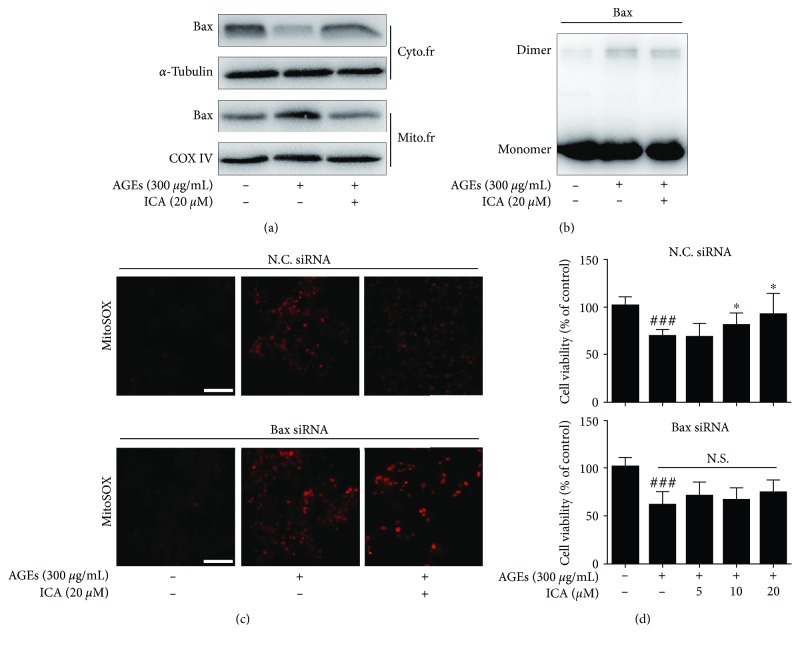
ICA regulated the biological activity of Bax. (a) ICA inhibited Bax transfer to mitochondria. (b) Bax dimer was detected by chemical cross-linking technology. (c and d) The reverse effects of Bax gene silencing on ICA-mediated antioxidative stress and neuroprotection were detected using MitoSOX and MTT assay. Scale bar = 50 *μ*m. Data were presented as mean ± S.D. from independent experiments performed in triplicate. ^###^*P* < 0.001 relative to the control group; ^∗^*P* < 0.05 relative to the model group. N.S., not significant.

Bax is a proapoptotic protein, which comes from Bcl-2 family. Normally, Bax exists in the cytoplasm; however, Bax could homooligomerize under stress conditions and translocate from the cytoplasm to the mitochondrial outer membrane, further causing mitochondrial dysfunction subsequently [31, 32]. Our study found that ICA could obviously inhibit Bax accumulation on the mitochondrial outer membrane. In addition, the formation of the Bax dimer was blocked by ICA (Figures 6(a) and 6(b)). This indicated that ICA could inhibit mitochondrial apoptosis by regulating the target protein Bax. Finally, we sought to elucidate whether the existence of Bax was essential for ICA for its antiapoptotic role. As shown in Figures 6(c) and 6(d), the effects of ICA-mediated neuroprotection and antioxidative stress were markedly reversed by knocking down Bax with a specific siRNA. This result suggested that Bax acted as a key target responsible for an ICA-dependent antiapoptotic effect.

## 4. Discussion

Diabetic encephalopathy (DE) is one of the most severe complications of diabetes, which can be manifested as cognitive impairment. It has also been acknowledged that diabetes is one of the independent risk factors for cognitive dysfunction. Pathological studies have shown that the brains of DE patients have the following pathological features similar to Alzheimer's disease (AD): amyloid deposition and abnormal phosphorylation of tau protein [33]. What's more, DE and AD share many mechanisms of pathogenesis, such as insulin resistance, cytotoxicity of AGEs, oxidative stress, and inflammation. Therefore, some scholars recently presented a hypothesis that AD is a kind of type 3 diabetes [34–36].

In our experiments, we studied one of the common pathogenic factors of DE and AD, the advanced glycation end products (AGEs). AGEs are a crucial product of nonenzymatic catalysis with the aldehyde group of glucose and the amino group of protein, which shows cytotoxicity. During normal aging, AGEs may exist in different cells. But in patients with diabetes and AD, the accumulation of AGEs in cells significantly increases. In patients with type 2 diabetes, hyperglycemia accelerates the accumulation of AGEs, which are widely found in the kidneys, retinal vessels, and central nervous system [37, 38]. The combination of AGEs with its receptor RAGE can activate a variety of proinflammatory cytokines and produce a large number of reactive oxygen species (ROS), which can damage mitochondrial function and ultimately lead to cell apoptosis. At present, it has been found that the increase of AGEs can cause the hyperphosphorylation of tau protein and the accumulation of A*β*, thus accelerating the progress of cognitive impairment and neurodegeneration [39].

Icariin (ICA) is the main active ingredient extracted from the Chinese herbal medicine *Epimedium brevicornu* Maxim. ICA has a variety of activities, such as neuroprotection, antitumor, antioxidation, immune regulation, and promotion of reproduction [40–42]. In terms of neuroprotection, our research group studied the neuroprotective effect of ICA on A*β*_25-35_-induced cytotoxicity and its related potential mechanism. The results indicated that ICA significantly increased cell viability and inhibited the apoptosis rate. A mechanism study showed that ICA could inhibit A*β*_25-35_-induced tau protein hyperphosphorylation via the PI3K/Akt/GSK-3*β* signaling pathway [43]. In addition, our research group also explored the protective effect of ICA on learning and memory in an ionic channel level. The results suggested that ICA exerted a protective effect due to the effect of accommodating voltage-gated Ca^2+^ channels (VGCCs) and N-methyl D-aspartate (NMDA) receptor channels. This effect may facilitate the long-term potentiation (LTP) [44]. Since DE and AD have many common pathogenic factors, pathological features, and pathological mechanisms, this experiment further explored whether ICA also had a protective effect on DE. In addition, the model used in our study is AGE-induced PC12 cells.

Apoptosis is a programmed cell death. It plays a crucial role in cell death and has been widely studied for neuroprotective agent development [45]. In this study, we found that the viability of PC12 cells obviously decreased after the induction of AGEs, while ICA could significantly improve the cell survival and inhibit LDH release in a concentration-dependent manner, which confirmed the neuroprotective effect of ICA. In addition, Hoechst 33258 and AO/EB staining showed that AGE stimulation induced the fragmented or shrunken nuclei, which was effectively inhibited by ICA by regulating the caspase-9/3 signaling pathway. All these indicated that ICA could inhibit PC12 cell apoptosis.

Oxidative stress can promote apoptosis. Under normal circumstances, oxidative stress and antioxidant stress are in a state of dynamic equilibrium. However, when stimulated by some risk factors, including AGEs and OGD/R, excessive reactive oxygen radicals (ROS) could be produced in cells [16, 46]. Excessive ROS can increase mitochondrial membrane permeability, allowing the release of cytochrome C into the cytoplasm, followed by the activation of the mitochondria-dependent caspase signaling pathway, which ultimately leads to apoptosis. In addition, malondialdehyde (MDA) is the product of the lipid peroxidation of polyunsaturated fatty acids on a cell membrane triggered by ROS, and superoxide dismutase (SOD) is an important substance for removing ROS in vivo. Previous studies demonstrated that AGEs induced cell apoptosis which could be blocked by NAC, a ROS scavenger [47, 48]. Similar to the effect of NAC, our result showed that ICA could attenuate endogenous and mitochondrial ROS by reducing MDA production and increasing SOD activity. All of these suggested that ICA is a neuroprotective agent with a significant antioxidant effect.

Drug targets are the major biological basis for treating various diseases. Thus, it is important for deeply interpreting the pharmacological action mechanism of traditional Chinese medicine (TCM) by exploring the targets of the active ingredients of TCM [49]. In this study, we constructed ICA beads to identify the direct pharmacological target proteins [50, 51]. The target of ICA was also identified as Bax, a proapoptotic regulator of the Bcl-2 family. We also used the methods of SPR, CETSA, and DARTs to confirm that ICA could specifically bind to the target protein Bax. Western blot showed that ICA could not only inhibit the transformation of Bax from the cytoplasm to mitochondria under AGE insult but also inhibit the formation of a Bax dimer. When the target protein Bax was knocked down by siRNA, the antiapoptotic activity of ICA was significantly blocked and its antioxidant activity was obviously weakened as well. These observations indicated that Bax was an important target protein for ICA to exert a neuroprotective effect.

## 5. Conclusions

Taken together, ICA could specifically bind to the target protein Bax to inhibit the formation of a Bax dimer and its migration to mitochondria, thereby reducing the production of endogenous ROS induced by AGEs, improving antioxidant capacity, and exerting an antiapoptotic effect. This study accurately analyzed the pharmacological mechanism of ICA against apoptosis from the source of the pharmacological target, thereby interpreting the neuroprotective effect of ICA. In addition, this study also identified a potential new target for the treatment of DE.

## Data Availability

The data used to support the findings of this study are available from the corresponding author upon request.

## Abbreviations

AGEs: Advanced glycosylation end products
Apaf-1: Apoptosis activating factor-1
AO/EB: Acridine orange/ethidium bromide
AD: Alzheimer's disease
CETSA: Cellular thermal shift assay
DARTS: Drug affinity responsive target stability
DCFH-DA: 2,7-Dichlorofluorescein diacetate
DE: Diabetic encephalopathy
DM: Diabetes mellitus
DMEM: Dulbecco's modified eagle medium
FBS: Fetal bovine serum
ICA: Icariin
LDH: Lactate dehydrogenase
LTP: Long-term potentiation
MDA: Malondialdehyde
MTT: 3-(4,5-Dimethylthiazol-2-yl)2,5-diphenyltetrazolium bromide
mtPTP: Mitochondrial permeability transition pore
NMDA: N-methyl D-aspartate
OGD/R: Oxygen-glucose deprivation/reperfusion
PVDF: Polyvinylidene fluoride
RAGE: Receptor of advanced glycosylation end products
ROS: Reactive oxygen species
SOD: Superoxide dismutase
SPR: Surface plasmon resonance
VGCCs: Voltage-gated Ca^2+^ channels.
